# Does acupuncture the day of embryo transfer affect the clinical pregnancy rate? Systematic review and meta-analysis

**DOI:** 10.5935/1518-0557.20180057

**Published:** 2018

**Authors:** Juan-Enrique Schwarze, Juan Pablo Ceroni, Carolina Ortega-Hrepich, Sonia Villa, Javier Crosby, Ricardo Pommer

**Affiliations:** 1 Unidad de Medicina Reproductiva Clinica Las Condes, Santiago, Chile; 2 Departamento OB/GIN, Universidad de Santiago, Santiago, Chile; 3 Unidad de Medicina Reproductiva Clinica Monteblanco, Santiago, Chile

**Keywords:** acupuncture, IVF, meta-analysis

## Abstract

The effects of acupuncture on IVF outcomes is still unknown. We carried out a systematic review and meta-analysis of RCT to determine whether acupuncture performed at the time of ET improves outcomes. We searched Medline and Embase from January 1990 to June 2017, for the following terms): (acupuncture; acupuncture therapy) and (reproductive techniques, assisted; *in vitro* fertilization; embryo transfer). We selected RCT that compared acupuncture with sham acupuncture or no treatment. We included only trials in which acupuncture involved the insertion of needles into traditional meridian points. We evaluated the methodological quality of the trials using the Cochrane risk of bias tool. The measure of treatment effect was the pooled odds ratio of achieving a clinical pregnancy, ongoing pregnancy, or live birth for women in the acupuncture group compared with women in the control group. For pooled data, summary test statistics were calculated using the Mantel-Haenszel method, using the Rev-Man software, version 5.1. We analyzed six studies, including 2,376. In all trials, there were no significant differences between the groups concerning the mean numbers of embryos transferred, the mean age of the women undergoing the procedure, diagnose and use of ICSI. Acupuncture performed the day of ET was associated with a reduced risk of clinical pregnancy (0.87, 95% confidence interval 0.77 to 0.98). The pooled rate difference was -0.06 (-0.12 to -0.01) for clinical pregnancy. None of the trials reported significant adverse effects of acupuncture.

## INTRODUCTION

Approximately 10% of couples in reproductive age suffer from infertility. For many of these couples, *in vitro* fertilization with embryo transfer (IVF-ET) provides the best possibility to achieve pregnancy. However, only 20% of initiated cycles and 35% of embryo transfers result in the delivery of a healthy baby (Zegers-Hochschild *et al.*, 2016a;b). Therefore, repeated treatment cycles are needed to achieve pregnancy. The need for repetition, not only place significant economic pressure on the couple, but it is also invasive and time-consuming.

After achieving blastocyst development, implantation is the factor with the greatest limitation on IVF-ET. Governed by complex mechanisms, the interaction between embryo and endometrium depends on the quality of each. New therapies that can improve this process are highly desirable.

As an important part of traditional Chinese medicine, acupuncture has gained increased popularity worldwide due to its convenience, lack of side effects, and unique therapeutic effects. Acupuncture has been used in China for centuries to regulate the female reproductive system (Chang *et al.*, 2002). As a method of treating disease, the theory of acupuncture is based on the energy flow of qi and the principles of traditional Chinese medicine meridians and acupoints. It is said that the imbalance of qi in the body causes disease, which can be treated by stimulating specific acupoints on the body surface.

Some potential mechanisms for its effects on fertility have been postulated (Chang *et al.*, 2002). Acupuncture may stimulate blood flow to the uterus by inhibiting uterine central sympathetic nerve activity (Stener-Victorin *et al.*, 1996), and may stimulate the production of endogenous opioids, which may inhibit the central nervous system outflow and the biological stress response (Yang *et al.*, 2008).

However, the effect of acupuncture on IVF outcomes is still unknown. Since the first relevant clinical research was published in 1999 a growing number of studies have been performed to explore the therapeutic effects of acupuncture on the outcomes of IVF-ET (Stener-Victorin *et al.*, 1999). These studies had variable designs and generally yielded inconclusive or conflicting results, rendering the clinical decision of whether to recommend or not the use of acupuncture during IVF difficult.

We conducted a systematic review and meta-analysis of randomised controlled trials to determine whether acupuncture performed at the time of ET improves the outcome among women undergoing IVF ET.

## METHODS

### Identification of studies

We searched the computerised databases Medline and Embase from January 1990 to June 2017. We used the following terms as free text terms and MeSH terms (shown in italics): (*acupuncture; acupuncture therapy*) and (*reproductive techniques, assisted; fertilization in vitro; embryo transfer*). We also searched the list of references of relevant publications.

### Selection criteria, data extraction and quality assessment

We selected randomised controlled trials that compared acupuncture with sham acupuncture or no treatment. Because we were evaluating acupuncture as a complement to embryo transfer, we considered only trials in which acupuncture was administered within one day of the procedure, with the objective of improving success rates. Trials that included intracytoplasmic injection of sperm as well as *in vitro* fertilisation were eligible. We excluded trials that evaluated other interventions in conjunction with acupuncture.

For trials to be eligible, we had to be able to extract data on at least one of the following outcomes, as recommended: clinical pregnancy (that is, presence of at least one gestational sac or fetal heartbeat, confirmed by transvaginal ultrasound), ongoing pregnancy (that is, pregnancy beyond 12 weeks of gestation, as confirmed by fetal heart activity on ultrasound), or live birth. We included only trials in which acupuncture involved the insertion of needles into traditional meridian points. The needles could be inserted into tender points in addition to the traditional meridian points.

We excluded trial with electrical stimulation of the needles. We also excluded trials of laser acupuncture and electro-acupuncture without needle insertion, because most authorities believe acupuncture involves needle insertion (Smith *et al.*, 2012).

We imposed no restrictions on publication type (that is, either full article or abstract), and restricted the language to English. Two authors (JPC and JES) independently selected articles and extracted data, with disagreements resolved by discussion.

We evaluated the methodological quality of the trials using the Cochrane risk of bias tool. The items evaluated were: concealment of allocation of randomisation sequence (selection bias), allocation concealment (selection bias), blinding of participants and personnel (detection bias), incomplete outcome data (attrition bias), selective reporting (reporting bias) and other biases (Higgins *et al.*, 2011).

### Data synthesis and analysis

The measure of treatment effect was the pooled odds ratio of achieving a clinical pregnancy, ongoing pregnancy, or live birth for women in the acupuncture group compared with women in the control group. For pooled data, summary test statistics were calculated using the Mantel-Haenszel method, via the Rev-Man software, version 5.1.

Our meta-analyses were based on the number of women randomised (rather than on the number of treatment attempts-that is, cycles of *in vitro* fertilisation) with the intention-to-treat approach analysis.

### Subgroup analysis

We evaluated heterogeneity using the I^2^ test (Higgins *et al.*, 2003), which indicates the proportion of variability across trials not explained by chance alone, and the *p*-value of the X^2^ test of heterogeneity. Although interpreting the importance of inconsistency depends on other factors in addition to the I^2^ values (e.g. *p*-value from X^2^ test, magnitude and direction of effects), the Cochrane Handbook suggests the following rough guide to interpreting the I^2^ values: low, moderate, and high to *I*^2^ values of 25%, 50%, and 75%, respectively (Higgins *et al*., 2003).

If the overall I^2^ value for all trials was reduced when we separated the trials into subgroups according to source of bias, we would use the subgroup results as primary. Otherwise, the pooled results from all trials would be used for our primary analysis, but with the results from the two subgroups also present.

## RESULTS

**Figure 1:**
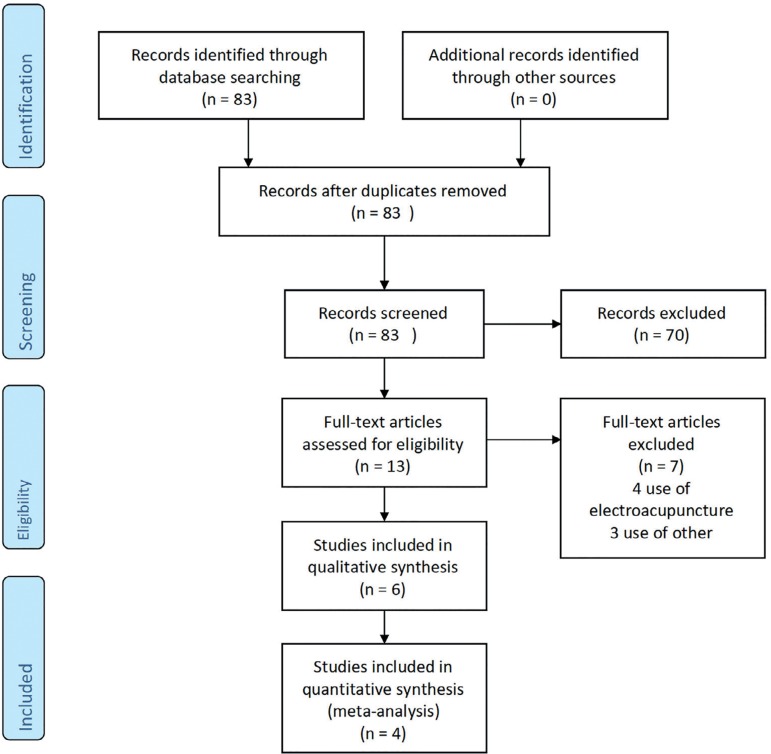
Flow chart of the study selection process for eligible studies in the systematic review

Figure 1 shows details of the selection process. Six randomized controlled trials, with 2,376 participants, met the selection criteria. All trials were published in English since 2009, and conducted in three different Western countries (Paulus *et al.*, 2002; Westergaard *et al.*, 2006; Domar *et al.*, 2009; Andersen *et al.*, 2010; Moy *et al.*, 2011) and China (So *et al.*, 2009). They were all published as full reports.

All six trials used a pragmatic design, including typical clinical populations and using typical interventions before and after randomization. All included a broad selection of women undergoing *in vitro* fertilisation, with a wide range of ages, diagnostic categories of infertility, durations of infertility, and numbers of previous treatment cycles.

The timing of the acupuncture sessions relative to embryo transfer differed somewhat among the trials (Table 1). However, in all the trials the women received acupuncture immediately before or immediately after the embryo transfer.

**Table 1:** Summary of studies included.

Study	Country	Intervention	Results
Paulus *et al.*, 2002	Germany	80 patients underwent 25 minutes of acupuncture before and after ET; the control were 80 patients without acupuncture	34/80 got pregnant in the acupuncture group, versus 21/80 in the control group
Westergaard *et al.*, 2006	Denmark	95 patients underwent 25 minutes of acupuncture before and after ET; the control were 87 patients without acupuncture	40/95 got pregnant in the acupuncture group, versus 24/87 in the control group
Domar *et al.*, 2009	USA	78 patients underwent 25 minutes of acupuncture before and after ET; the controls were 68 patients without acupuncture	39/78 got pregnant in the acupuncture group, versus 29/68 in the control group
So *et al.*, 2009	China	185 patients underwent 25 minutes of acupuncture before and after ET; the controls were 185 patients with sham acupuncture	81/185 got pregnant in the acupuncture group, versus 102/185 in the control group
Andersen *et al.*, 2010	USA	314 patients underwent 30 minutes of acupuncture before and after ET; the control were 321 patients with sham acupuncture	126/314 got pregnant in the acupuncture group, versus 149/321 in the control group
Moy *et al.*, 2011	USA	86 patients underwent 25 minutes of acupuncture before and after ET; the controls were 74 patients with sham acupuncture	39/86 got pregnant in the acupuncture group, versus 39/74 in the control group

In all trials, the acupuncture protocol and selection of acupuncture points were designed for the sole purpose of improving pregnancy rates.

### Risk of bias of included studies

**Figure 2:**
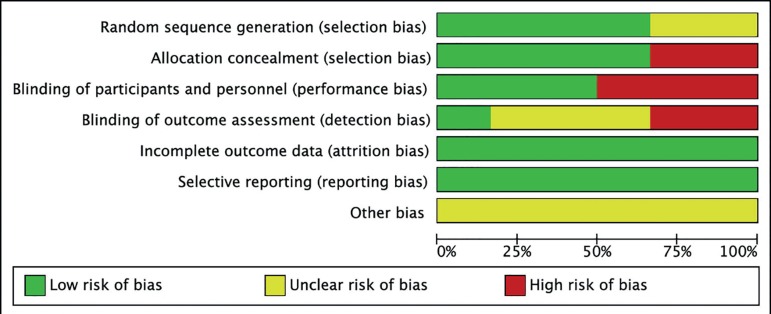
Risk of bias graph

**Figure 3:**
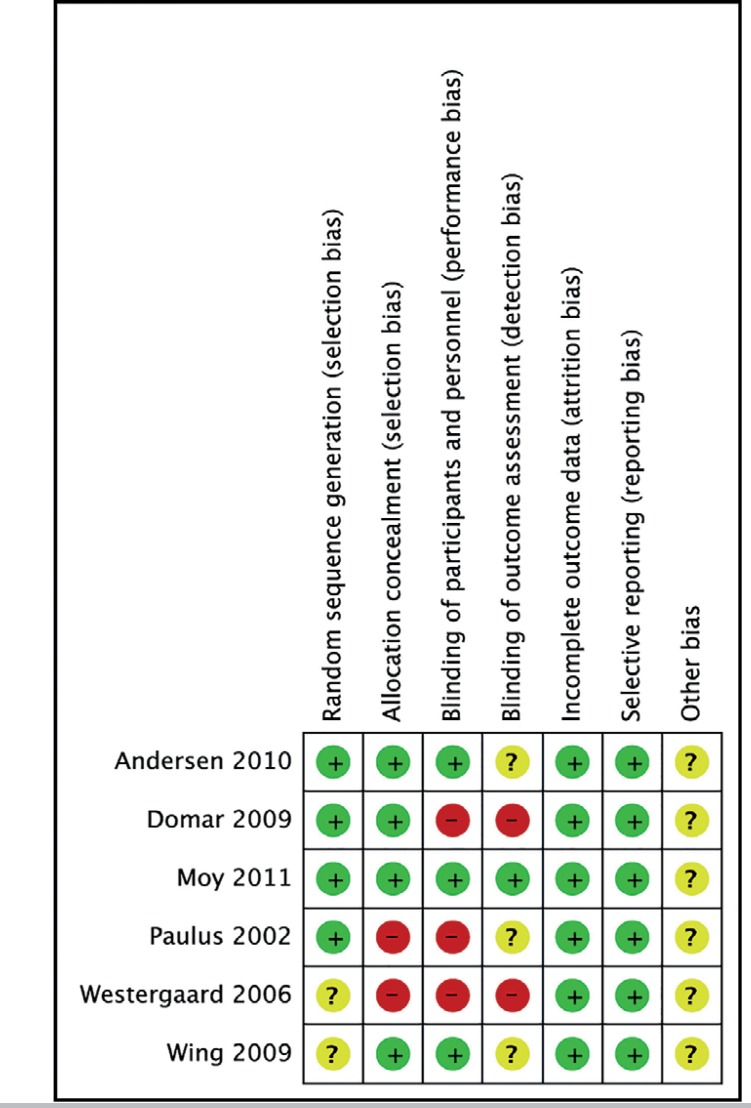
Risk of bias summary

The trials generally had high internal validity, in terms of randomisation procedures and follow-up of participants. In all trials, the investigators confirmed no losses to follow-up, which is usual for *in vitro* fertilisation cycles (Figures 2 and 3).

Four trials used sham acupuncture controls (Paulus *et al.*, 2002; So *et al.*, 2009; Andersen *et al.*, 2010; Moy *et al.*, 2011); one trial used needles that penetrated the skin at acupuncture points selected not to influence fertility (Andersen *et al.*, 2010), and three used non-penetrating sham needles (Paulus *et al.*, 2002; So *et al.*, 2009; Moy *et al.*, 2011). In all trials, the acupuncture sessions lasted 25-30 minutes (Table 1).

**Figure 4:**
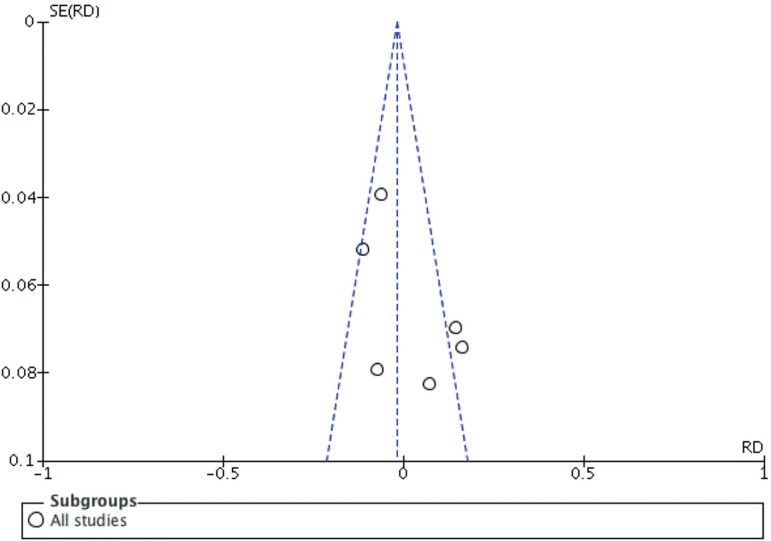
Funnel plot analysis

In all trials, there were no significant differences between the groups in the mean numbers of embryos transferred, mean age of the women undergoing the procedure, diagnose and use of ICSI. Funnel plot analyses showed that there were nonpublication biases (Figure 4).

### Efficacy analysis

**Figure 5:**
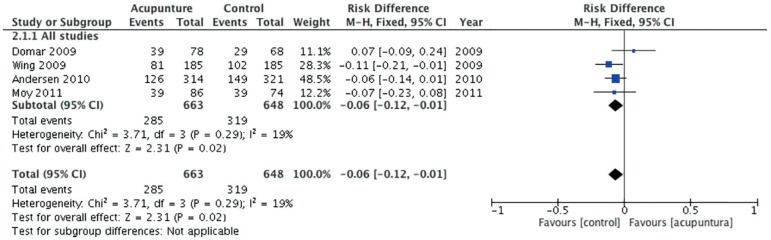
Forest plot of comparison: Acupuncture versus control, outcome: Clinical pregnancy per embryo transfer

The heterogeneity among studies was high, I^2^ 69% *p*-value=0.006; therefore, we restricted to those four randomised trials with less than 50% bias risk (Domar & Alper, 2013). Acupuncture performed the day of ET was associated with a reduced risk of clinical pregnancy (0.87, 95% confidence interval 0.77 to 0.98), (Figure 5). The pooled rate difference was -0.06 (-0.12 to -0.01) for clinical pregnancy.

None of the trials reported significant adverse effects of acupuncture.

## DISCUSSION

After the meta-analysis of the studies with a low-risk of bias, we found that acupuncture performed on the day of ET decreases significantly the risk of achieving a pregnancy. Probably, the main strength of our study is that we randomised controlled trials with low risk of bias, and low heterogeneity. Nevertheless, the main weakness is that there are few studies dealing with acupuncture at the time of ET, and we could not find any study published after 2011.

Other studies have dealt with these questions (Qian *et al.*, 2017; Manheimer *et al.*, 2008; 2013). Qian *et al*. (2017) ran a meta-analysis involving 30 studies, including 6,344 women, and found an improvement in the OR of clinical pregnancy of 1.26 (95% confidence interval, 1.06-1.50). However, they compared any accepted form of acupuncture, and did not evaluate the risk of bias among the studies. Manheimer *et al.* (2008) published a meta-analysis of seven trials, including 1,366 women, and found a significant improvement in the OR of pregnancy of 1.5 (95% confidence interval 1.27-2.14); however, they analysed studies with or without any adjuvant therapy, thus adding a source of bias to the study. Later, in 2013, the same group published a new meta-analysis of 16 trials, with a total of 4,021 women, and found no difference in the OR of pregnancy (1.65; 95% confidence interval 0.96-1.31), again the use of any adjuvant therapy was not a cause of exclusion (Manheimer *et al.*, 2013).

According to our findings, physicians should encourage their patients to avoid undergoing acupuncture on the day of ET, since it diminishes the chances of getting pregnant. It still has to be elucidated the mechanisms by which acupuncture negatively affects embryo implantation. So far, no evidence of a significant effect of acupuncture on vascular biomarkers and well-being (Phy *et al.*, 2017), endometrial and subendometrial vascularity, serum cortisol (So *et al.*, 2009)concentration and anxiety level has been demonstrated.

In conclusion, acupuncture performed on the day of ET has a significant effect on embryo implantation, however, a negative one. Therefore, clinicians should encourage their patients to avoid this technique the day of ET.
